# Fluorescent Analogues of Human α-Calcitonin Gene-Related Peptide with Potent Vasodilator Activity

**DOI:** 10.3390/ijms21041343

**Published:** 2020-02-17

**Authors:** Jing Zhu, Mahdieh Dagina Pedersen, Laraib Sabbah Ahmed, Bahareh Abdolalizadeh, Anne-Sofie Grell, Jais Oliver Berg, Peter Waaben Thulstrup, Henrik Franzyk, Lars Edvinsson, Anette Sams, Majid Sheykhzade, Paul Robert Hansen

**Affiliations:** 1 Department of Drug Design and Pharmacology, University of Copenhagen, Universitetsparken 2, 2100 Copenhagen, Denmark; zoe2120@outlook.com (J.Z.); mahdieh.dagina@yahoo.com (M.D.P.); sabbah.ahmed@gmail.com (L.S.A.); bahareh.abdolalizadeh@sund.ku.dk (B.A.); henrik.franzyk@sund.ku.dk (H.F.); mash@sund.ku.dk (M.S.); 2 Department of Clinical Experimental Research, Glostrup Research Institute, Rigshospitalet Glostrup, Nordstjernevej 42, 2600 Glostrup, Denmark; anne-sofie.jagd.grell@regionh.dk (A.-S.G.); lars.edvinsson@regionh.dk (L.E.); anette.nielsen.03@regionh.dk (A.S.); 3 Department of Plastic and Reconstructive Surgery, Herlev-Gentofte Hospital, University of Copenhagen, 2730 Herlev, Denmark; Jais.Oliver.Berg@regionh.dk; 4 Department of Chemistry, University of Copenhagen, Universitetsparken 5, 2100 Copenhagen, Denmark; pwt@chem.ku.dk

**Keywords:** human α-calcitonin gene-related peptide, 5(6)-carboxyfluorescein, solid-phase peptide synthesis, concentration-response curves

## Abstract

Human α-calcitonin gene-related peptide (h-α-CGRP) is a highly potent vasodilator peptide that belongs to the family of calcitonin peptides. There are two forms of CGRP receptors in humans and rodents: α-CGRP receptor predominately found in the cardiovascular system and β-CGRP receptor predominating in the gastrointestinal tract. The CGRP receptors are primarily localized to C and Aδ sensory fibers, where they are involved in nociceptive transmission and migraine pathophysiology. These fibers are found both peripherally and centrally, with extensive perivascular location. The CGRP receptors belong to the class B G-protein-coupled receptors, and they are primarily associated to signaling via Gα proteins. The objectives of the present work were: (i) synthesis of three single-labelled fluorescent analogues of h-α-CGRP by 9-fluorenylmethyloxycarbonyl (Fmoc)-based solid-phase peptide synthesis, and (ii) testing of their biological activity in isolated human, mouse, and rat arteries by using a small-vessel myograph setup. The three analogues were labelled with 5(6)-carboxyfluorescein via the spacer 6-aminohexanoic acid at the chain of Lys^24^ or Lys^35^. Circular dichroism (CD) experiments were performed to obtain information on the secondary structure of these fluorescently labelled peptides. The CD spectra indicated that the folding of all three analogues was similar to that of native α-CGRP. The three fluorescent analogues of α-CGRP were successfully prepared with a purity of >95%. In comparison to α-CGRP, the three analogues exhibited similar efficacy, but different potency in producing a vasodilator effect. The analogue labelled at the N-terminus proved to be the most readily synthesized, but it was found to possess the lowest vasodilator potency. The analogues labelled at Lys^35^ or Lys^24^ exhibited an acceptable reduction in potency (i.e., 3–5 times and 5–10 times less potent, respectively), and thus they have potential for use in further investigations of receptor internalization and neuronal reuptake.

## 1. Introduction

Human α-calcitonin gene-related peptide (h-α-CGRP) is a naturally occurring neuropeptide (ACDTATCVTHRLAGLLSRSGGVVKNNFVPTNVGSKAF-NH_2_), consisting of 37 amino acids and a disulfide bond between residues 2 and 7. The peptide is found in primary afferent sensory nerves, and it is widely distributed throughout the central and peripheral nervous systems in the body [1]. The structure of CGRP is highly conserved across species. The two isoforms of CGRP in humans and rodents (i.e., α- and β-CGRP) display >90% sequence homology, differing only by one and three amino acids in rats and humans, respectively [2]. The CGRP receptor family has been reviewed recently [3]. Structure-activity studies of h-α-CGRP have shown that the disulfide-bonded loop (i.e., residues 2–7) is required for receptor activation. Furthermore, h-α-CGRP consists of an α-helix (residues 8–18), a region incorporating a β-bend (residues 19–26), and a C-terminal portion (residues 27–37) that is characterized by bends between residues 28–30 and 33–34 [4]. The CGRP receptor is involved in various diseases such as hypertension, ischemic heart disease, and migraine [5]. Targeting CGRP receptors may be a useful approach for therapeutic intervention [6], and CGRP-neutralizing antibodies have recently been developed for the prevention of migraine [7]. Furthermore, h-αCGRP (8–37)-amide antagonistic peptides have proved to be interesting therapeutic ligands [8], and hence numerous analogues have been reported [9]-. Human α-CGRP linked to a fatty acid with improved half-life has also been described [10].

Previous studies have shown that the released CGRP from perivascular afferent sensory nerve fibers is subjected to neuronal uptake in guinea pig perivascular nerves of the basilar artery [11] as well as in rat dura mater and mouse vas deferens [12,13]. These findings have contributed to a better understanding of the role of CGRP in health and disease. Nevertheless, the detailed cellular and molecular mechanisms for (re)uptake of CGRP into the sensory nerve terminals (see Figure S15) and the receptor trafficking inside the cell remain unclear; in these cases fluorescent h-α-CGRP analogues will be useful for investigating the mode of action. As part of an ongoing study on the mode of action for h-α-CGRP-induced vasodilation, we here present a 9-fluorenylmethyloxycarbonyl (Fmoc)-based strategy for the solid-phase synthesis (SPPS) of h-α-CGRP and three fluorescent analogues. Thus, four peptides were synthesized by Fmoc SPPS: h-α-CGRP without tagging (i.e., wild-type CGRP) and three fluorescent analogues labelled with 5(6)-carboxyfluorescein (CF) via a 6-aminohexanoic acid (Ahx) spacer either at the N-terminus or on the side chain of Lys^24^ or Lys^35^, all containing the native disulfide bond. Following SPPS and characterization, the biological activity of these peptides was tested in isolated mesenteric arteries of rat and mice, as well as in human subcutaneous arteries.

## 2. Results and Discussion

### 2.1. Synthesis of Fluorescent CGRP Derivatives

**Figure 1:**
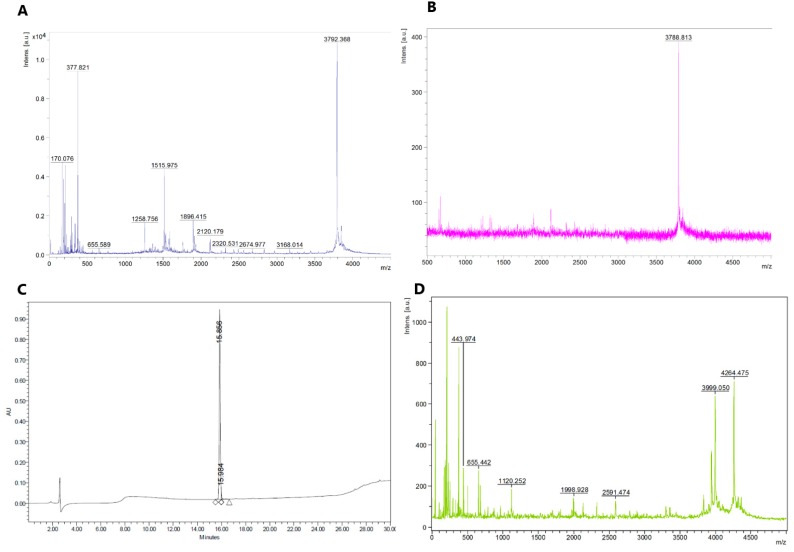

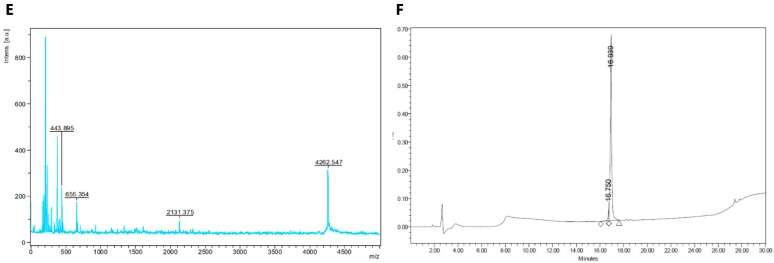
MALDI-TOF-MS and analytical HPLC of human α-calcitonin gene-related peptide (h-α-CGRP) (**1**) and [Lys^35^(Ahx-CF)] h-α-CGRP (**3**). (**A**) MALDI-TOF-MS of h-α-CGRP before oxidation and purification; (**B**) MALDI-TOF-MS and (**C**) analytical HPLC of h-α-CGRP (**1**) after oxidation and purification; (**D**) MALDI-TOF-MS of [Lys^35^(Ahx-CF)] h-α-CGRP (**3**) before oxidation and purification; (**E**) MALDI-TOF-MS and (**F**) analytical HPLC of [Lys^35^(Ahx-CF)] h-α-CGRP (**3**) after oxidation and purification. Calculated masses: reduced CGRP, 3791.373; oxidized CGRP, 3789.357 Da; reduced [Lys^35^(Ahx-CF)] h-α-CGRP, 4262.838 Da; oxidized [Lys^35^(Ahx-CF)] h-α-CGRP, 4260.822 Da.

**Scheme 1:**
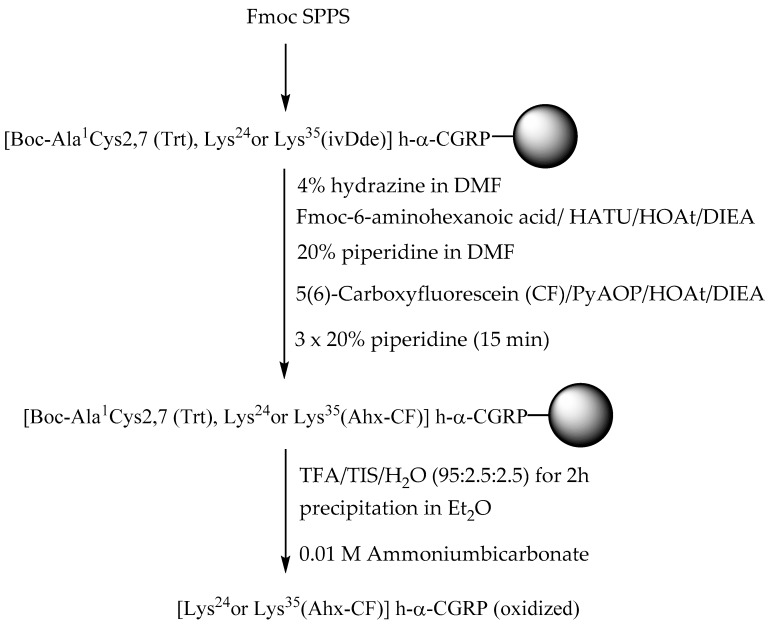
**9**-Fluorenylmethyloxycarbonyl (Fmoc) solid-phase synthesis (SPPS) strategy for synthesizing fluorescent analogues of h-α-CGRP.

The aim of this work was to develop a method for SPPS of fluorescently labelled CGRP analogues; the strategy is outlined in Scheme 1. The peptide h-α-CGRP (**1**) was synthesized by standard Fmoc chemistry, on a ChemMatrix resin, equipped with a peptide amide linker (PAL) [14]. Activation of the Fmoc-protected amino acid building blocks was carried out by using 1-[bis(dimethylamino)methylene]-1H-1,2,3-triazolo[4,5-b]pyridinium 3-oxide hexafluorophosphate (HATU)/1-hydroxy-7-azabenzotriazole (HOAt)/diisopropylamine (DIEA). Trityl (Trt)-protected Fmoc-Cys(Trt))-OH was used for incorporation of residues 2 and 7, whereas pseudoproline dipeptides [15,16] were employed for incorporation of the residues in positions 5–6, 8–9, and 16–17 [10]. Fmoc deprotection was accomplished by treatment with 20% piperidine in dimethylformamide (DMF). The peptide was cleaved from the solid support with simultaneous removal of the permanent side-chain protecting groups by using trifluoroacetic acid (TFA)/H_2_O/triisopropylsilane (TIS) (90:2.5:2.5 *v*/*v*) for 2 h. The crude peptide was oxidized in 0.01 M NH_4_HCO_3_ and purified by preparative HPLC, and then characterized by analytical HPLC and MALDI-TOF-MS. The peptides **2** ([Lys^24^(Ahx-CF)] h-α-CGRP) and **3** ([Lys^35^(Ahx-CF)] h-α-CGRP) were synthesized as above with the following modifications: Fmoc-Lys(1-(4,4-Dimethyl-2,6-dioxo-cyclohexylidene)-3-methyl-butyl (ivDde))-OH was used for introduction of residue 24 or 35. Following coupling of the last amino acid building block (i.e., *tert*-butyloxycarbonyl (Boc)-Ala-OH), the ivDde temporary protecting group was cleaved by treatment with 2% hydrazine hydrate in DMF (3 × 15 min). After DMF wash, *N*-Fmoc-6-aminohexanoic acid (Fmoc-Ahx-OH) was coupled either to Lys^24^ or Lys^35^. Following Fmoc deprotection, CF was coupled overnight to the Ahx-elongated side-chain of Lys^24^ or Lys^35^ using (7-azabenzotriazol-1-yloxy)tripyrrolidinophosphonium hexafluorophosphate (PyAOP)/HOAt/DIEA, followed by treatment with 20% piperidine in DMF to cleave any phenolic esters formed. After cleavage from the resin, the crude linear peptide was subjected to disulfide bond formation, and final purification was performed as described above for the native peptide. MALDI-TOF-MS and analytical HPLC data of h-α-CGRP and [Lys^35^(Ahx-CF)] h-α-CGRP are shown in Figure 1. For **4** ([*N*^α^ (Ahx-CF)] h-α-CGRP), the synthesis was concluded with Fmoc-Ala-OH followed by derivatization of the N-terminus as decribed above.

Fluorescently labelled peptides have a wide range of applications in flow cytometry, fluorescence microscopy, and immunofluorescence-based assays [17]. Typically, peptides and proteins are labelled with fluorescein-type moieties via amino or thiol groups of the side chains of lysine or cysteine residues [18]. Alternatively, the N-terminal amino group can be tagged. CF has to be activated before use, whereas fluorescein isothiocyanate (FITC) and carboxyfluorescein *N*-succinimidylester (CFSE) are activated fluorescein derivatives [19,20]. However, FITC has limited use in Fmoc SPPS, as acidic conditions are often required for cleavage of the peptide from the linker. It has been shown that N-terminal FITC-labelled peptides undergo degradation during TFA treatment by a mechanism similar to the Edman degradation, leading to the formation of a byproduct consisting of fluorescein attached to the N-terminal amino acid [21]. In comparison to FITC and CFSE, 5(6)-carboxyfluorescein is much cheaper and more efficiently introduced [22]. Furthermore, the latter is fully compatible with Fmoc SPPS [23]. Fluorescein is known to exist in three forms: lactonic, zwitterionic, and quinoid structures [24]. The free carboxyl-group in position 2 of fluorescein determines its fluorescence properties, and this group in 5(6)-carboxyfluorescein can form an intramolecular lactonic bridge, which serves as an auto-protection, because of the steric hindrance [19]. In comparison to the two fluorescent analogues labelled at Lys^24^ (**2**) and Lys^35^ (**3**), wild-type CGRP and the analogue labelled at the N-terminus (i.e., **4**) proved easier to synthesize and to be have better solubility properties. The main reason for the low yield obtained for the analogues labeled at Lys^24^ and Lys^35^ is the incomplete removal of the ivDde group (+206 Da; see Figure 1D), because the ivDde-containing peptide is difficult to separate from the fluorescent analogues. In our first synthesis of [Lys^24^(Ahx-CF)] h-α-CGRP and [Lys^35^(Ahx-CF)] h-α-CGRP, we used the standard conditions (i.e., 2% hydrazine in DMF; 3 × 15 min) for deprotection of the ivDde protecting group [25]. However, we found that the deprotection of the ivDde group was incomplete, and increasing the hydrazine concentration to 4% had some effect, but not to a satisfactory degree. The problem of incomplete ivDde protection has been reported in the literature, and 3–5% hydrazine in DMF has been recommended [26,27,28].

We investigated an alternative strategy (Figure S7) in which the two fluorescent analogues labelled at Lys^24^ and Lys^35^ were synthesized by using the acid-labile 4-methyltrityl (Mtt) group as the temporary lysine protecting group. The Mtt group is typically cleaved with 1% TFA in dichloromethane (DCM) or with the weakly acidic 30% hexafluoroisopropanol in DCM. A potential problem with this strategy is that the Cys(Trt) residue may be deprotected. However, Cys(Trt) requires 7–10% TFA in DCM [29]. Furthermore, 30% hexafluoroisopropanol in DCM for Mtt removal has been reported to be compatible with Cys(Trt) protection [30].

The Mtt approach resulted in approximately the same amount of product after purification.

**Figure 2:**
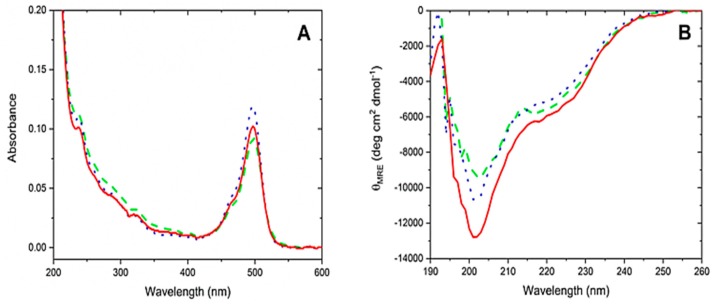
Spectroscopic data for fluorescent conjugates of CGRP in acetonitrile (ACN–High potassium Physiological Saline Solution (KPSS) (1:20, v/v) at 25 °C. (**A**) UV-VIS absorbance used for concentration determination. (**B**) UV-CD (Ultraviolet- Circular Dichroism) spectra for comparison of folding. [N-terminal(Ahx-CF)] h-α-CGRP (―), [Lys^24^(Ahx-CF)] h-α-CGRP (----), [Lys^35^(Ahx-CF)] h-α-CGRP (····).

In Figure 2A the visible absorption spectra of α-CGRP analogues displaying the fluorescent labelling at Lys^24^, Lys^35^, or at the N-terminus are shown (as recorded in aqueous buffer containing 5% acetonitrile). The absorbance maximum is seen at 498 nm, which is slightly redshifted as compared to the unconjugated CF. In Figure 2B, the far UV circular dichroism (CD) spectra of the analogues are shown (with the mean residue ellipticity as the *y*-axis). The spectra of the three analogues resemble each other within experimental uncertainty for both absorbance and CD. The CD curve displays a shoulder at 220 nm and a stronger negative band at 200 nm, which is indicative of a secondary structure comprising both α-helical folding and random coil. The mean residue ellipticity signal at 222 nm was of a magnitude of ≈6000–7000 deg·cm^2^·dmol^−1^, which is in fair agreement with values previously reported for wild-type α-CGRP [31,32]. It is known that the α-CGRP folding is very dependent on solvent composition, and membrane-mimicking co-solvents have been shown to confer a dramatically increased α-helicity [31,32,33].

Simms et al. reported the synthesis of [Lys^15^(CF)] human α-CGRP, which was used for detection of photoaffinity cross-linking between calcitonin receptor-like receptor (CLR) with receptor-activity-modifying protein 1 (RAMP). The cAMP (Cyclic adenosine monophosphate) production on HEK293T cells transfected with CLR and RAMP and exposed to the [Lys^15^(CF)] analogue was investigated. The analogue showed more than a 10-fold loss of activity, but was found to be a full agonist [34]. In continuation of this work, the same authors reported a fluorescent analogue, prepared by click chemistry of [propargylglycine^3^] h-α-CGRP and tetramethylrh (odamine (TAMRA) azide (tetramethylrhodamine 5-carboxamido-(6-azidohexanyl)) to give [TAMRA^3^]-h-α-CGRP. Concentration-response curves of cAMP production at CGRP receptors expressed in COS-7 cells revealed similar activity of the analogue. Furthermore, the authors showed that [TAMRA^3^]-h-α-CGRP is internalized with CGRP receptors expressed in HEK2935 cells [35].

### 2.2. Biological Activity of Three Fluorescently Tagged CGRP Analogues

**Figure 3:**
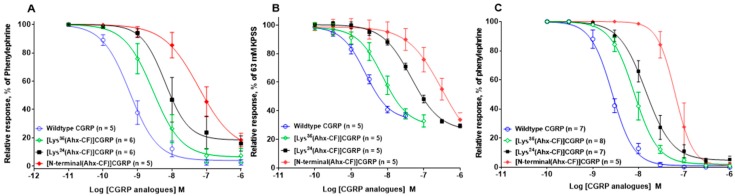
Cumulative concentration–response curves (10 pM–1 µM) for wild-type CGRP and fluorescently tagged analogues in isolated (**A**) mouse mesenteric arteries with an average lumen diameter of 212 ± 14 µm (*n* = 22), (**B**) rat mesenteric arteries with an average lumen diameter of 284 ± 8 µm (*n* = 20), and (**C**) human subcutaneous arteries with an average lumen diameter of 654 ± 39 µm (*n* = 27). Data points represent mean values and vertical bars indicate ± SEM (Standard error of mean), where this value exceeds the size of symbol (*n* = number of animals or number of human subcutaneous arterial ring segments). Relative responses are given as fraction (stated as percentages) of the initial vessel contractile response to either 10 µM phenylephrine or KPSS buffer containing 63 mM K^+^ just before they were challenged with wild-type h-α-CGRP and its fluorescently tagged analogues.

α-CGRP and its fluorescent analogues induced concentration-dependent vasodilator effects in isolated arterial segments of all three species (mouse, rat, and human; Figure 3A–C). The maximal effects were similar, but the potencies differed as compared to that of wild-type α-CGRP (Table 1). In all species, wild-type α-CGRP is the most potent agonist, followed by fluorescent analogues labelled at Lys^35^ and Lys^24^, whereas the N-terminally tagged analogue proved to be the least potent. 

In human subcutaneous arteries, all compounds induced concentration-dependent vasodilation with similar maximal effects, but with different potency (Figure 3C), Thus, in comparison to native h-α-CGRP, [Lys^35^(Ahx-CF)] h-α-CGRP is approximately 3-fold less potent, whereas [Lys^24^(Ahx-CF)] h-α-CGRP is approximately 5-fold less potent, and [N-terminal(Ahx-CF)] h-α-CGRP is approximately 24-fold less potent. The vasodilator responses, induced by these compounds in isolated mouse and rat mesenteric arteries (Figure 3A,B), followed the same order of potency: wild-type α-CGRP > [Lys^35^(Ahx-CF)] h-α-CGRP > [Lys^24^(Ahx-CF)] h-α-CGRP > [N-terminal(Ahx-CF)] h-α-CGRP. In rodents (rats and mice), [Lys^35^(Ahx-CF)] CGRP is around 3- to 4-fold less potent than wild-type CGRP, whereas [Lys^24^(Ahx-CF)] h-α-CGRP is approximately 15- to 19-fold less potent, and [N-terminal(Ahx-CF)] h-α-CGRP is approximately 70- to 128-fold less potent, respectively. 

Generally, wild-type h-α-CGRP and its three fluorescent analogues induced comparable vasodilator responses in isolated arteries from all three species (mouse, rat, and human) with similar efficiency but with different potency. Because potency depends on both affinity and effect, the observed activity of the peptides proved to be similar, and hence these four peptides appear to possess different binding affinities for the CGRP receptor. This is somehow expected, as tagging of the peptide may induce conformational/structural modifications leading to changes in affinity for the receptor. However, the potency assessed in functional studies depends on both affinity and efficacy [36]. In functional studies, especially when dealing with complex receptors such as G-protein-coupled receptors (GPCRs), the potency of the ligand can be affected by intracellular signaling (i.e., efficiency of receptor–effector coupling and stimulus–response amplification). However, further studies are needed to verify this. Moreover, our data show that the N-terminal part of the peptide is the most important for high affinity, as fluorescent tagging at the N-terminus of h-α-CGRP conferred the most pronounced loss of potency across all three species. Previous studies have also emphasized the functional importance of the N-terminal part by showing that its extension by a single tyrosine residue or a biotin moiety leads to a decrease in the potency of the peptide by 10–40-fold and 150-fold, respectively [1,4,37].

In all three species, there was no statistically significant difference between the potency of peptides displaying the label at Lys^35^ and Lys^24^. However, our data show a narrower gap between the potency of fluorescent analogues with label at Lys^35^ and Lys^24^ in human subcutaneous arteries as compared to that found for rodent arteries. This may be explained by the fact that Lys^24^ in h-α-CGRP is surrounded by different amino acid residues, as seen in rat-αCGRP, thereby stabilizing a more favorable α-helical structure needed for interaction with human CGRP receptors.

The synthesized h-α-CGRP (wild-type CGRP) and h-α-CGRP purchased from BACHEM showed almost overlapping concentration-response curves (Figure S14).

## 3. Materials and Methods

Disposable 5 mL polypropylene reactors fitted with a polytetrafluoroethylene (PTFE) filter were acquired from Thermo Scientific, Waltham, Massachusetts, USA TFA, piperidine, and Fmoc-protected L-amino acids were purchased from Iris-Biotech GmbH, Marktredwitz, Germany PAL ChemMatrix, DIEA and triisopropylamine were from Sigma-Aldrich, (St Louis, MO, USA). 1-Hydroxy-7-azabenzotriazole (HOAt), 1-[bis(dimethylamino)methylene]-1H-1,2,3-triazolo[4,5-b]pyridinium 3-oxide hexafluorophosphate (HATU), and 7-azabenzotriazol-1-yloxy)tripyrrolidinophosphonium hexafluorophosphate (PyAOP)) were from GL Biochem Shanghai, China. Dimethylformamide (DMF; synthesis grade), dichloromethane (DCM; optical grade), and acetonitrile (ACN; optical grade) were from VWR (Copenhagen, Denmark). All reagents and solvents were used without further purification. Human- αCGRP, Fmoc-Ala-Thr(ψMe,Mepro)-OH, (Fmoc-Val-Thr(ψMe,Mepro)-OH), and ((Fmoc-Leu-Ser(ψMe,Mepro)-OH)) were purchased from BACHEM (GmbH, Rhein, Germany). Phenylephrine hydrochloride (PE) was purchased from Sigma-Aldrich (St Louis, MO, USA). Stock solutions of the compounds (1 or 10 mM) were stored frozen in small aliquots at −20 °C, and dilutions were prepared just before carrying out the experiments.

Physiological salt solution (PSS) had the following composition (in mM): NaCl 119, NaHCO_3_ 25, KCl 4.7, CaCl_2_ 1.5, KH_2_PO_4_ 1.18, MgSO_4_·7H_2_O 1.17, ethylenediaminetetraacetic acid (EDTA) 0.027, and glucose 5.5, with pH adjusted to 7.4. High potassium Physiological Saline Solution (KPSS) was prepared by replacing all sodium with an equimolar amount of potassium, resulting in a total K^+^ concentration of 125 mM. The above reagents were obtained from Sigma-Aldrich (St Louis, MO, USA).

### 3.1. Peptide Synthesis

Peptide synthesis was performed manually on a PAL ChemMatrix resin (0.48 mmol/g) in a syringe equipped with a fritted filter. Coupling of Fmoc-protected amino acids were done using HATU, HOAt, and DIEA (4:4:8 eq) in DMF for 2 h. For residue 24 or 35, Fmoc-Lys (ivDde)-OH was used. Fmoc removal was accomplished with 20% piperidine in DMF (3 × 4 min). The N-terminal amino acid Ala^1^ was coupled as Boc-Ala-OH (to obtain **2** and **3**) and Fmoc-Ala-OH to obtain **4**. Pseudoprolines were used in position 5–6 (Fmoc-Ala-Thr(ψMe,Mepro)-OH), 8–9 ((Fmoc-Val-Thr(ψMe,Mepro)-OH)), and 16–17 ((Fmoc-Leu-Ser(ψMe,Mepro)-OH)). The ivDde protecting group was removed with 4% hydrazine in DMF (3 × 15 min). The spacer Fmoc-6-amino hexanoic acid was coupled as described above.

The fluorescent probe 5(6)-carboxyfluorescein was coupled using PyAOP (12 equiv), HOAt (12 equiv), and DIEA (24 equiv), as described by Stahl [19]. After coupling for 48 h, the resin was treated with 20% piperidine in DMF for 30 min. The resin was then drained and washed with DMF, DCM, and ethanol (EtOH) and lyophilized. Following synthesis, the product was cleaved from the resin with TFA/H_2_O/TIS (95:2.5:2.5), precipitated in ether, and lyophilized. The peptides were oxidized using 0.01 M aqueous ammoniumbicarbonate for 48 h.

Peptide purification was achieved by a preparative reverse-phase HPLC system consisting of a Waters 600 Pump, In-line Degasser, 600 Controller, and 2996 Photodiode Array Detector. The column used was a Waters XSelect Peptide CSH C18 OBDTM, 5 µm, 19 mm × 250 mm with H_2_O/ACN gradient. Fractions were concentrated and lyophilized. An analytical reverse-phase HPLC system was used to determine purity of the product, consisting of a Waters 717 plus Autosampler, In-line Degasser AF, 600 Controller, and 2996 Photodiode Array Detector; the column used was a Waters Symmetry C18, 5 µm, 4.6 mm × 250 mm on an H_2_O/ACN gradient. The products were characterized by matrix-assisted laser desorption/ionization time-of-flight mass spectrometry (Bruker Microflex) using α-cyano-4-hydroxycinnamic acid as matrix.

Peptide-resins were typically cleaved in 100 mg portions and resulted in the following yield after HPLC-purification (ivDde approach): wild-type CGRP (6.0 mg), [Lys^35^(Ahx-CF)] h-α-CGRP (2.6 mg), [Lys^24^(Ahx-CF)] h-α-CGRP (5.4 mg), and [N-terminal(Ahx-CF)] h-α-CGRP (6.3 mg). Mtt (approach): [Lys^35^(Ahx-CF)] h-α-CGRP (3.4 mg) and [Lys^24^(Ahx-CF)] h-α-CGRP (3.0 mg).

### 3.2. Animals

Male NMRI mice (30–35 g, Taconic Europe) and male Sprague-Dawley rats (250–300 g, Taconic Europe, Lille Skensved, Denmark) were housed in a local animal facility in a temperature- and humidity-controlled environment with 12 h light and 12 h dark cycle and ad libitum access to standard chow and water. All animal procedures were strictly within national laws and guidelines and the Guide for the Care and Use of Laboratory Animals was followed. Animals were first sedated with CO_2_ and then euthanized by guillotination followed by subsequent exsanguination. The mesenteric arcade was immediately excised and pinned out in a silicon-covered Petri dish. Afterwards, the second order mesenteric arteries were isolated and immersed in ice-cold physiological saline solution (PSS) (see composition above). Average normalized lumen diameter (µm) of mesenteric arteries were 211 ± 14 (*n* = 22) and 284 ± 8 (*n* = 20) in mice and rat, respectively.

### 3.3. Human Subcutaneous Arteries

The study is approved by The Committees on Health Research Ethics in the Capital Region of Denmark (H-15005617) and carried out in accordance with The Code of Ethics of the World Medical Association (Declaration of Helsinki). After giving informed consent, adult subjects (3 females, age: 30–50 years-old and weight: 70–90 kg) donated a whole piece of abdominal skin with subcutaneous adipose tissue to the project. All patients were former obese subjects who had lost weight and underwent plastic surgery at the Department of Plastic Surgery V, Herlev Hospital, Denmark, with removal of excess skin and adipose tissue from the abdomen. After removal, the surgical specimen was immediately transferred to a +5 °C isotonic saline solution (9 g/L NaCl) and transported to our laboratory. In the laboratory, tissues were kept in oxygenated PSS at 4 °C, and subcutaneous resistance arteries were carefully dissected free of surrounding adipose tissue and were cut into 1–2 mm long ring segments. Average lumen diameter (µm) of human subcutaneous arteries was 654 ± 39 (*n* = 27).

Experimental protocol related to functional studies used a small vessel wire myograph setup.

Vasomotor properties of isolated artery segments were studied using a wire myograph that records isometric tension. Mesenteric arteries were mounted as ring segments (1–2 mm long) on two stainless steel wires (40 μm diameter) between the two jaws in the organ bath of a small vessel wire myograph (Danish MyoTechnology A/S, Aarhus, Denmark). One jaw was connected to a micrometer screw enabling adjustments of the distance between the wires. The other jaw was connected to a force displacement transducer attached to an analogue-digital converter unit (ADInstruments, Chalgrove, United Kingdom). Vascular tone and evoked force-development were recorded using a Power Lab unit (LabChart 7, ADInstruments) [38]. Each segment was immersed in 37 °C bicarbonate buffer solution (physiological saline solution (PSS)), which was continuously aerated with 5% CO_2_/95% O_2_ resulting in pH of 7.4. The vessels were then stretched to 90% of the normal internal circumference that each vessel would have under a passive transmural pressure of 100 mmHg, thereby ensuring maximal force development. Following an equilibration period of approximately 20 min, each segment was stimulated thrice with KPSS (containing 125 mM K+), to examine the viability and reproducibility of contractions in the vessels. Vessels were accepted only if the maximal active pressure to KPSS (calculated according to the Laplace relation: ΔP_max_= [2 × ΔT_max_]/[internal diameter or lumen diameter]) exceeded 13.3 kPa [38]. Each vessel was pre-contracted with either 10 µM phenylephrine (human and mice) or 63 mM KPSS (bicarbonate buffer solution containing 63 mM K^+^) (rat) until a stable pre-contraction tone was achieved. Afterwards, cumulative concentration-response curves (with a half log increments) were performed using tagged and wild-type human-αCGRP (10 pM–1 µM). Choice of organ-baths in the experiments was randomized [38].

### 3.4. Data Analysis and Statistics

All vessel responses were measured as absolute values (Nm^−1^) in LabChart. Relaxations were then calculated as percentage of the pre-contraction tone (percentage relative responses) induced either by 10 µM phenylephrine in human and mice or by 63 mM KPSS in rat. Vessel wall tension (ΔT) = force/(vessel wall length), where vessel wall length = 2 × segment length. Active tension (ΔT) was determined by subtracting the passive tension (resting tension in PSS) from the total tension (T). All concentration–response curves are analyzed by iterative non-linear regression analysis using GraphPad Prism 8 program (GraphPad Corp, San Diego, CA, USA). Each regression line was fitted to a sigmoid equation: E/E_max_ = A[M]^nH^/(A[M]^nH^ + IC_50_[M]^nH^), where E_max_ is the maximal response developed to the agonist; A[M] is the concentration of agonist; and n_H_ is a curve-fitting parameter, the Hill coefficient [10]. Sensitivity to agonists is expressed as pIC_50_ value, where pIC_50_ = −log (IC_50_ [M]), and IC_50_ [M] is the molar concentration of agonist required to produce half-maximal response. Differences between mean values were analyzed by one-way ANOVA followed by Bonferroni’s post-hoc multiple comparison test, accepting significance at *p* < 0.05. Data are presented as mean ± SEM (*n* = number of animals (i.e., one arterial ring segment per animal) or number of human subcutaneous arterial ring segments).

### 3.5. CD Spectroscopy

UV-VIS absorbance spectra were recorded on a Shimadzu UV-3600 UV-VIS-NIR (near infra-red) instrument using a 10 mm quartz cuvette. Circular dichroism (CD) spectra were recorded from 190 to 260 nm on a Jasco J-815 CD spectrometer set to 1 nm bandwidth and using a 1 nm step in continuous mode with a digital integration time of 4 s and a scanning speed 50 nm/min. The CD spectra were carried out in quartz cells with a pathlength of 1 mm, and were measured at room temperature. Corresponding reference spectra of pure solvent were recorded and subtracted for both CD and absorbance. 

Stock solutions of Lys^24^-, Lys^35^-, and N-terminally conjugated, fluorescent CGRP analogues were prepared by dissolving lyophilized peptide in 50% ACN/water with a concentration of ≈1 mg/mL. For CD, the solutions were diluted ×10 in PSS buffer. Thus, the final content of ACN was 5% (*v*/*v*). The actual concentrations of the fluorescent analogues were determined by visible absorption spectra using the molar absorbance at 492 nm for 5(6)-carboxyfluorescein (ε492 nm = 76,000 M^−1^·cm^−1^). Mean residue ellipticity values for CD spectra were based on the concentration calculated in this way, taking 37 amino acids into account for all three analogues.

## 4. Conclusions

The present study describes the successful synthesis of three different single-labeled fluorescent analogues of α-CGRP by Fmoc SPPS. The labelling of α-CGRP was performed by introducing a 5(6)-carboxyfluorescein probe at the N-terminus, Lys^24^, or Lys^35^ via a 6-aminohexanoic acid spacer. After the labeling, the biological activity of the analogues was assessed by obtaining concentration–response curves for the α-CGRP analogues on arterial segments isolated from three species (mouse, rat, and human). The functional experiments were carried out in order to determine whether tagging of the native h-α-CGRP (i.e., wild-type CGRP) would result in loss of potency. [Lys^35^(Ahx-CF)] h-α-CGRP and wild-type CGRP showed similar potency, whereas [Lys^24^(Ahx-CF)] h-α-CGRP showed approximately 10-fold less potency as compared to that of the wild-type α-CGRP. The N-terminally tagged α-CGRP analogue was the least potent compound in all three species. Finally, the potency gap between the Lys^24^ and Lys^35^ tagged analogues was significantly narrowed in human arteries as compared to that found in rodents. Thus, the analogues [Lys^24^(Ahx-CF)] h-α-CGRP and [Lys^35^(Ahx-CF)] h-α-CGRP were found to constitute promising tool compounds for further studies, for instance, of the mechanism for internalization of CGRP receptors in live tissue and neuronal reuptake of the α-CGRP peptide.

## Figures and Tables

**Table 1:** E_max_, Hill coefficient, pIC_50_ values, and potency ratios when comparing wild-type CGRP to the three fluorescently tagged CGRP analogues in three different species (mouse, rat, and human).

Biological Activity	Wild-Type CGRP	[N-terminal(Ahx-CF)] CGRP	[Lys^24^(Ahx-CF)] CGRP	[Lys^35^(Ahx-CF)] CGRP
**Mouse mesenteric artery**				
**E_max_, %**	99 ± 7 (*n* = 5)	87 ± 13 (*n* = 5)	82 ± 10 (*n* = 6)	94 ± 8 (*n* = 6)
**pIC_50_**	9.14 ± 0.18 (*n* = 5)	7.30 ± 0.23 (*n* = 5) ***	7.96 ± 0.38 (*n* = 6) *	8.57 ± 0.21 (*n* = 6)
**Potency ratio**	1	70	15	4
**Hill slope**	1.0 ± 0.2 (*n* = 5)	0.9 ± 0.3 (*n* = 5)	1.3 ± 0.5 (*n* = 6)	1.0 ± 0.3 (*n* = 6)
**Rat mesenteric artery**				
**E_max_, %**	67 ± 5 (*n* = 5)	66 ± 5 (*n* = 5)	74 ± 3 (*n* = 5)	70 ± 6 (*n* = 5)
**pIC_50_**	8.61 ± 0.06 (*n* = 5)	6.50 ± 0.50 (*n* = 5) ***	7.35 ± 0.05 (*n* = 5) *	8.13 ± 0.11 (*n* = 5)
**Potency ratio**	1	128	19	3
**Hill slope**	1.3 ± 0.3 (*n* = 5)	0.9 ± 0.5 (*n* = 5)	1.1 ± 0.1 (*n* = 5)	1.2 ± 0.4 (*n* = 5)
**Human subcutaneous artery**				
**E_max_, %**	100 ± 3 (*n* = 7)	99 ± 8 (*n* = 8)	96 ± 5 (*n* = 7)	99 ± 4 (*n* = 5)
**pIC_50_**	8.62 ± 0.06 (*n* = 7)	7.25 ± 0.16 (*n* = 8) ***	7.85 ± 0.06 (*n* = 7) ***	8.12 ± 0.12 (*n* = 5)
**Potency ratio**	1	24	5	3
**Hill slope**	1.7 ± 0.2 (*n* = 7)	2.0 ± 0.6 (*n* = 8)	1.5 ± 0.3 (*n* = 7)	1.6 ± 0.3 (*n* = 5)

Data are presented as mean ± SEM (*n* = number of animals or number of human subcutaneous arterial ring segments). In all three species, the maximal effects (E_max_) were not significantly different between the wild-type CGRP and its three fluorescently tagged analogues. The significance of differences in potencies when comparing wild-type CGRP with its three fluorescently tagged analogues is indicated by * *p* < 0.05, and *** *p* < 0.001.

## Supplementary Materials

Supplementary materials can be found at https://www.mdpi.com/1422-0067/21/4/1343/s1.

## Abbreviations

ACN	acetonitrile
Ahx	6-aminohexanoic acid (Ahx)
Boc	*tert*-butyloxycarbonyl
CD	circular dichroism
CF	5(6)-carboxyfluorescein
CFSE	carboxyfluorescein-N-succinimidylester
CLR	calcitonin receptor-like receptor
DCM	dichloromethane
DIEA	diisopropylamine
DMF	dimethylformamide
FITC	fluorescein isothiocyanate
Fmoc	9-fluorenylmethyloxycarbonyl
h-α-CGRP	human α-calcitonin gene-related peptide
HATU	1-[bis(dimethylamino)methylene]-1H-1,2,3-triazolo[4,5-b]pyridinium 3-oxide hexafluorophosphate
HOAt	1-hydroxy-7-azabenzotriazole
HPLC	high-performance liquid chromatography
ivDde	1-(4,4-Dimethyl-2,6-dioxo-cyclohexylidene)-3-methyl-butyl
MALDI-TOF-MS	matrix-assisted linear desorption ionization time-of-flight mass spectrometry
PyAOP	(7-azabenzotriazol-1-yloxy)tripyrrolidinophosphonium hexafluorophosphate)
Mtt	4-methyltrityl
SPPS	solid-phase peptide synthesis
TIS	triisopropylsilane
Trt	trityl
