# Supplementary material for: Fluorescent Analogues of Human α-Calcitonin Gene-Related Peptide with Potent Vasodilator Activity
Source: Int J Mol Sci. 2020 Feb 17;21(4):1343. doi: 10.3390/ijms21041343 (PMC7072916; doi:10.3390/ijms21041343)
Supplement: Supplementary file 1 [file ijms-21-01343-s001.pdf]

International Journal of
Molecular Sciences

MDPI

Supplementary Materials

S1: MALDI-TOF-MS of ([Lys^{24}(Ahx-CF)] h- -CGRP
before oxidation and purification.
S2: MALDI-TOF-MS of ([Lys^{24}(Ahx-CF)] h- -CGRP
after oxidation and purification.
S3: Analytical HPLC of ([Lys^{24}(Ahx-CF)] h- -CGRP
After oxidation and purification.
S4: MALDI-TOF-MS of ([*N* (Ahx-CF)] h- -CGRP)
before oxidation and purification
S5: MALDI-TOF-MS of ([*N* (Ahx-CF)] h- -CGRP)
after oxidation and purification
S6: Analytical HPLC of ([*N* (Ahx-CF)] h- -CGRP)
after oxidation and purification
S7: SCHEME: Synthesis of of ([Lys^{24}(Ahx-CF)] h- -CGRP and ([Lys^{35}(Ahx-CF)] h- -CGRP using Mtt for Lys protection.

S8: ([Lys^{24}(Ahx-CF)] h- -CGRP before oxidation and purification

S9: Analytical HPLC of ([Lys^{24}(Ahx-CF)] h- -CGRP
after oxidation and purification.
S10: MALDI-TOF-MS of ([Lys^{24}(Ahx-CF)] h- -CGRP
after oxidation and purification. Mtt was used for Lys^{24} protection
S11: MALDI-TOF-MS of ([Lys^{35}(Ahx-CF)] h- -CGRP
before oxidation and purification. Mtt was used for Lys^{35} protection.
S12: MALDI-TOF-MS of ([Lys^{35}(Ahx-CF)] h- -CGRP
after oxidation and purification. Mtt was used for Lys^{35} protection.
S13: Analytical HPLC of ([Lys^{35}(Ahx-CF)] h- -CGRP
after oxidation and purification. Mtt was used for Lys^{35} protection
S14: Concentration-response curves with compounds used in this study
S15: Show whole-mount staining of rat 2nd order mesenteric artery with the analogue [Lys^{35}(Ahx-CF)] h- -CGRP.

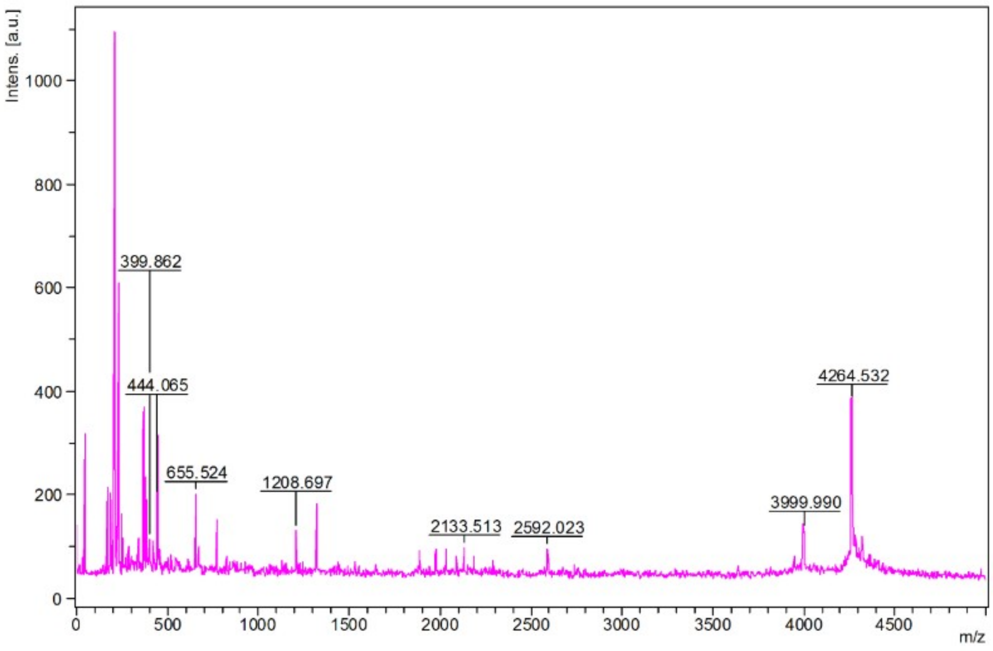

Figure S1. MALDI-TOF-MS of ([Lys[24](Ahx-CF)] h- -CGRP. Before oxidation and purification. IvDde was used for Lys[24] protection.

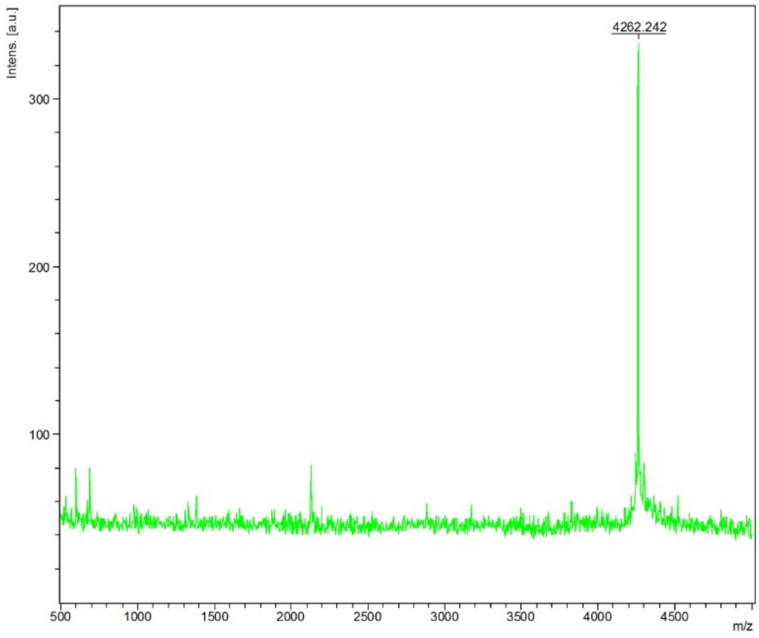

Figure S2. MALDI-TOF-MS of ([Lys[24](Ahx-CF)] h- -CGRP. After oxidation and purification. IvDde was used for Lys[24] protection.

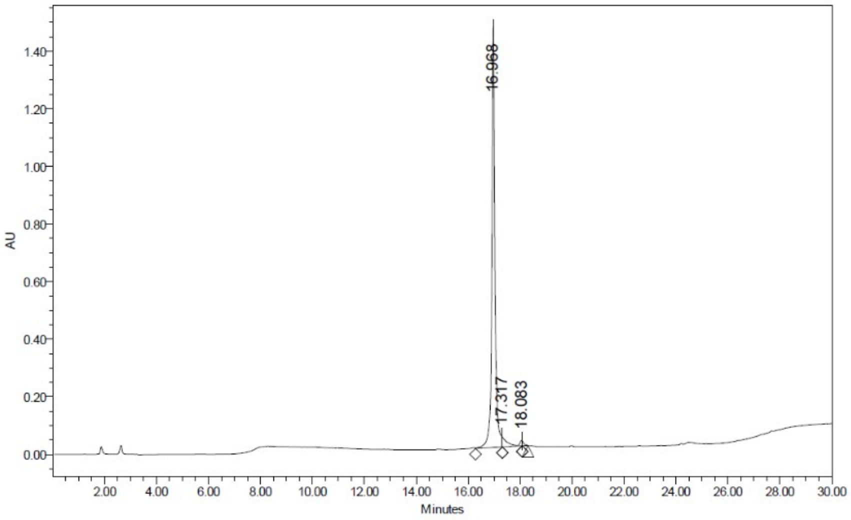

Figure S3. Analytical HPLC of ([Lys^{24}(Ahx-CF)] h- -CGRP. After oxidation and purification. IvDde was used for Lys^{24} protection.

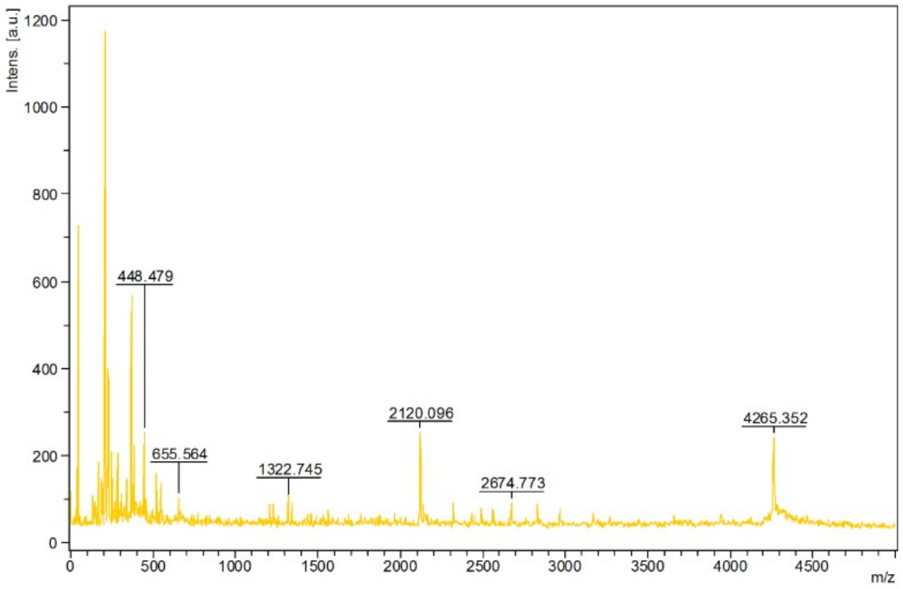

Figure S4. MALDI-TOF-MS of ([*N* (Ahx-CF)] h- -CGRP). before oxidation and purification.

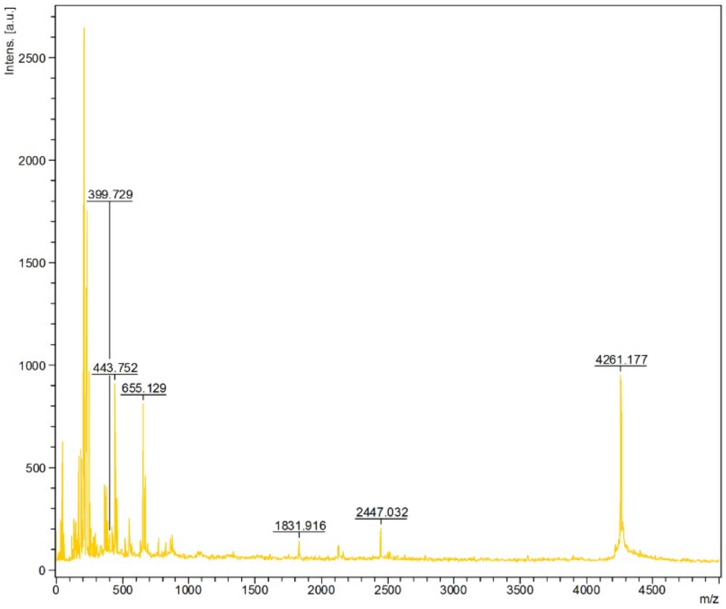

Figure S5. MALDI-TOF-MS of ([*N* (Ahx-CF)] h- -CGRP). after oxidation and purification.

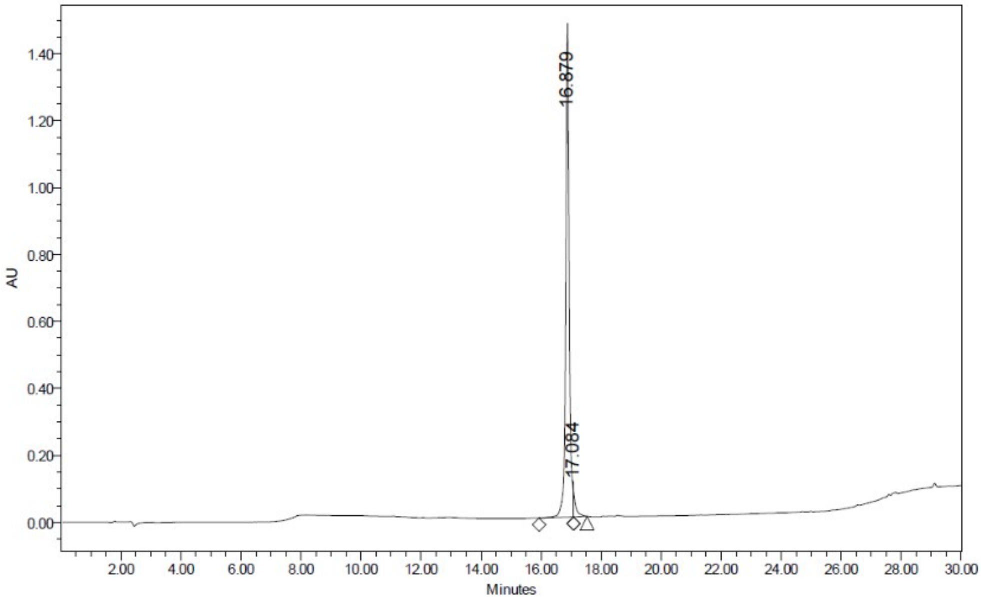

Figure S6. Analytical HPLC of ([*N* (Ahx-CF)] h- -CGRP). after oxidation and purification.

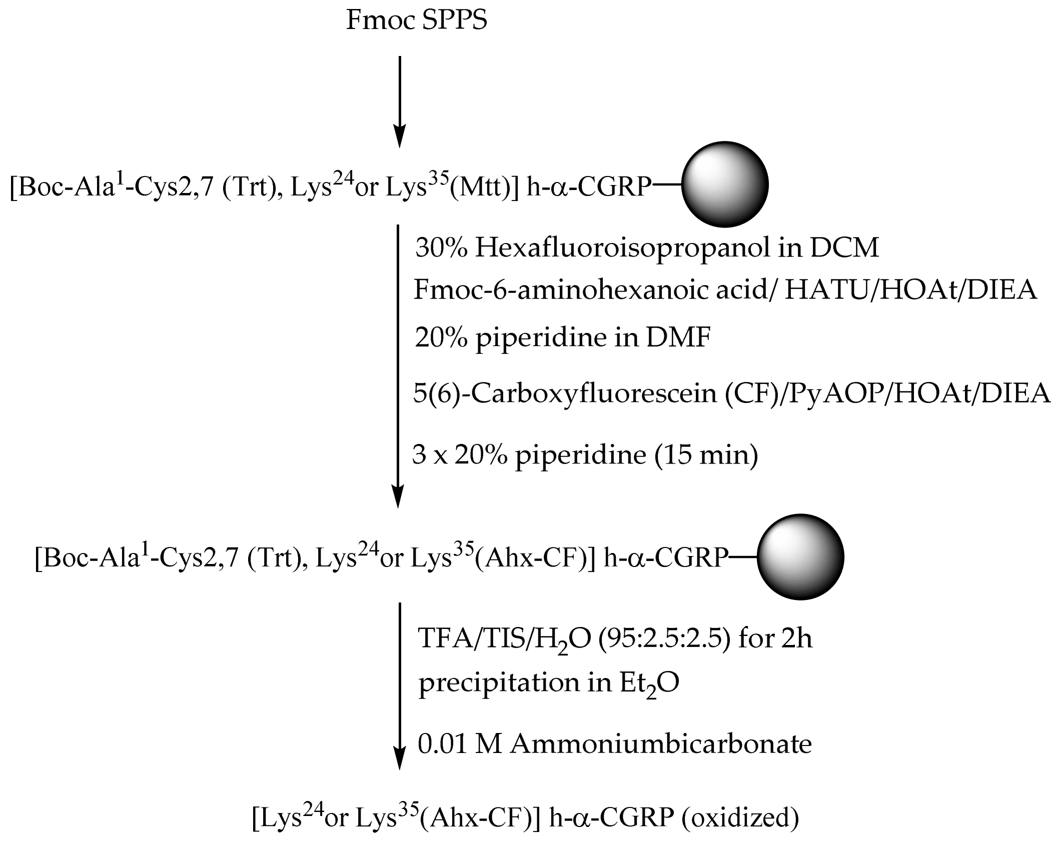

Figure 7. Syntesis of ([Lys24(Ahx-CF)] h- -CGRP and ([Lys35(Ahx-CF)] h- -CGRP using Fmoc-Lys(Mtt)-OH.

S7: SCHEME :

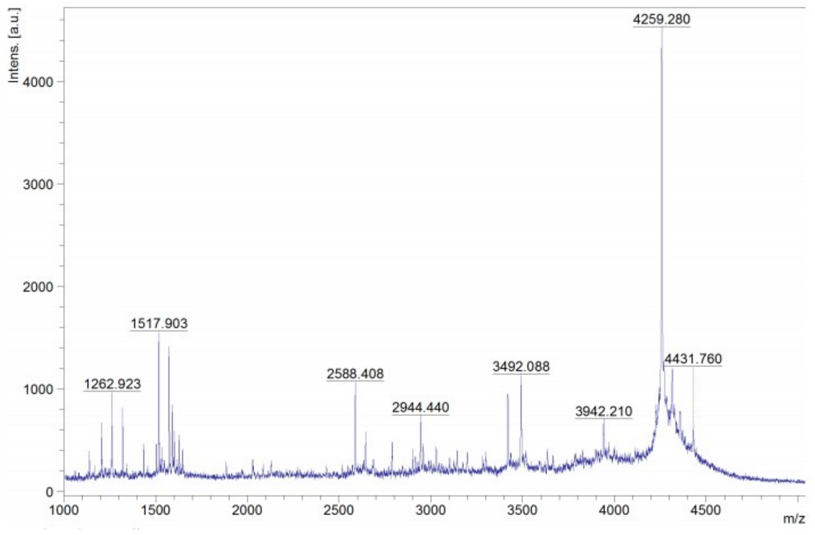

Figure S8. ([Lys^{24}(Ahx-CF)] h- -CGRP before oxidation and purification.

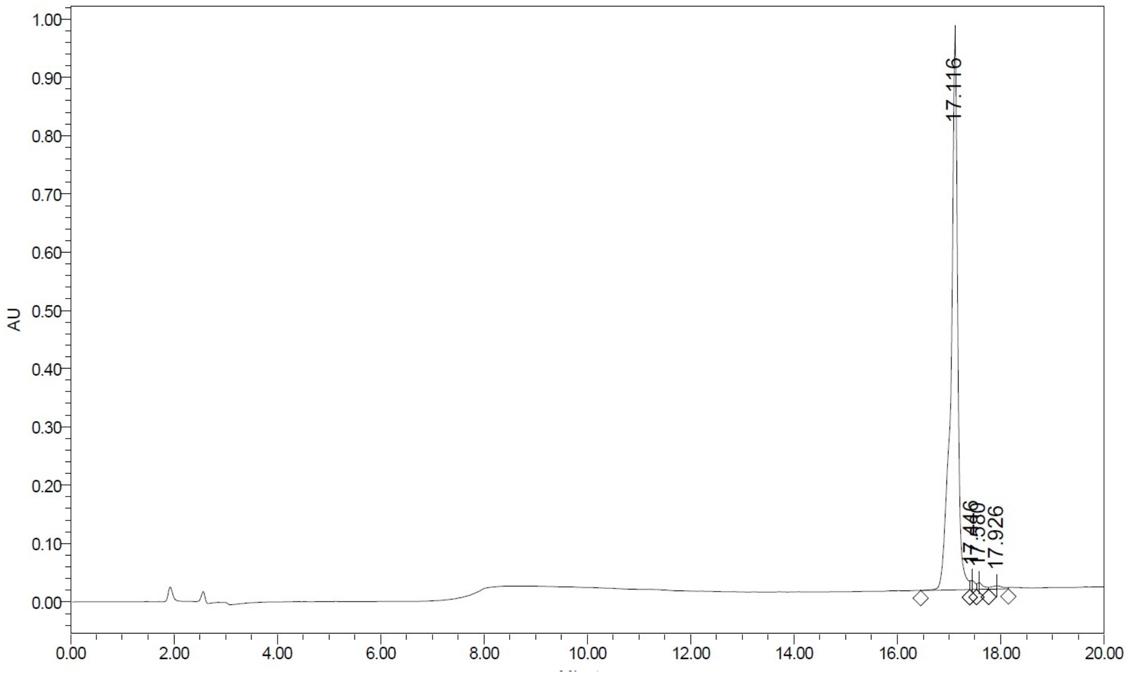

Figure S9. Analytical HPLC of ([Lys[24](Ahx-CF)] h- -CGRP after oxidation and purification. Mtt was used for Lys[24] protection.

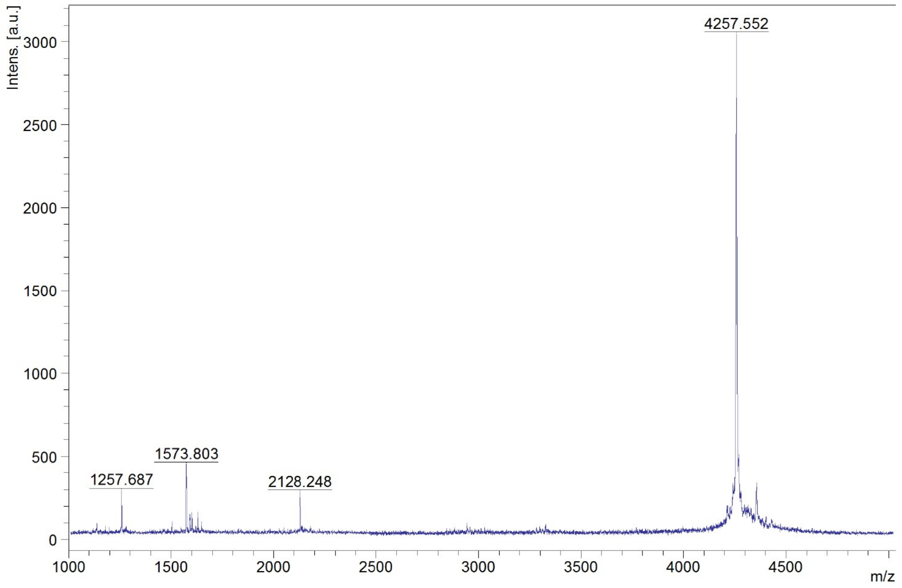

Figure S10. MALDI-TOF-MS of ([Lys[24](Ahx-CF)] h- -CGRP after oxidation and purification. Mtt was used for Lys[24] protection.

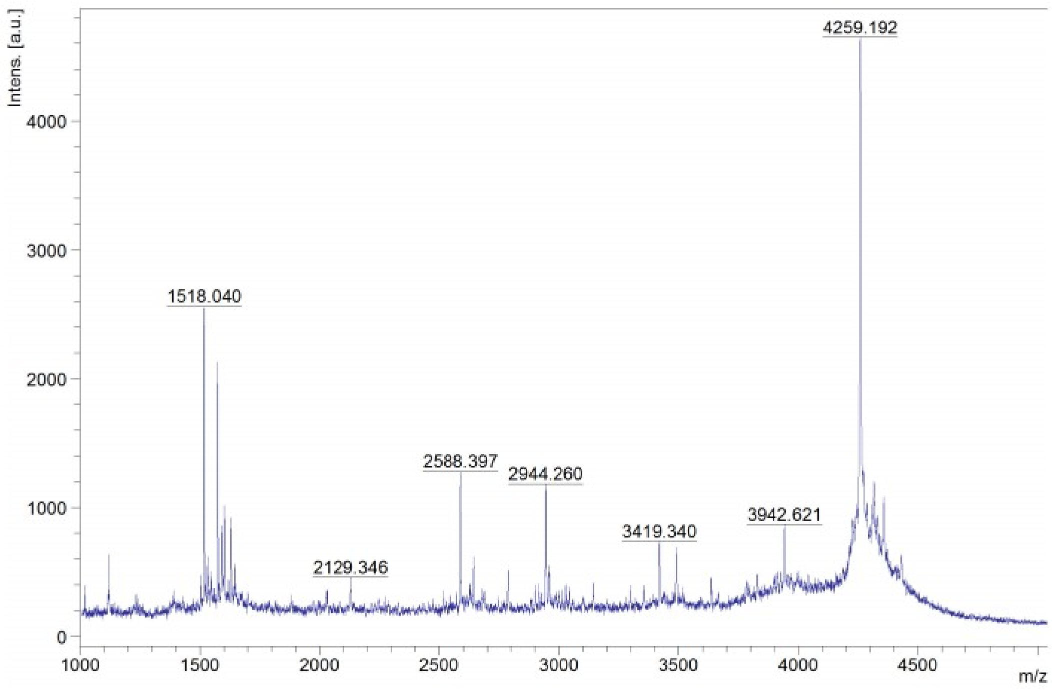

Figure S11. MALDI-TOF-MS of ([Lys[35](Ahx-CF)] h- -CGRP before oxidation and purification. Mtt was used for Lys[35] protection.

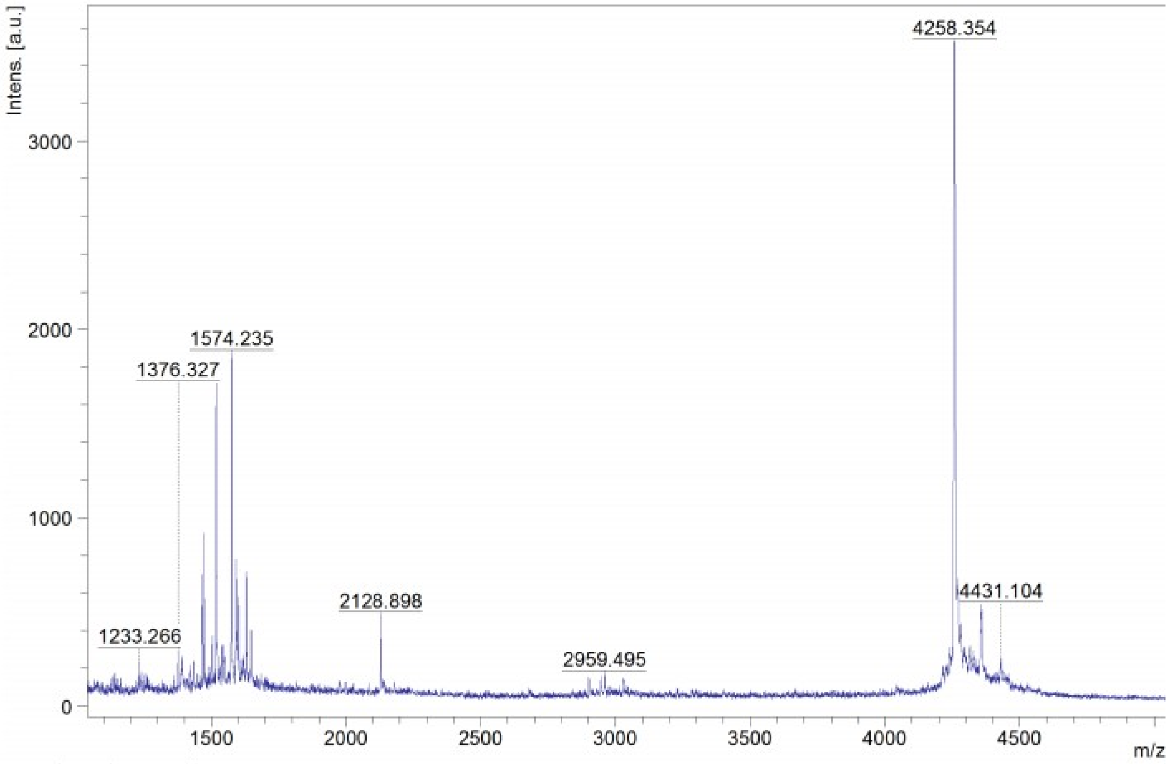

Figure S12. MALDI-TOF-MS of ([Lys[35](Ahx-CF)] h- -CGRP after oxidation and purification. Mtt was used for Lys[35] protection.

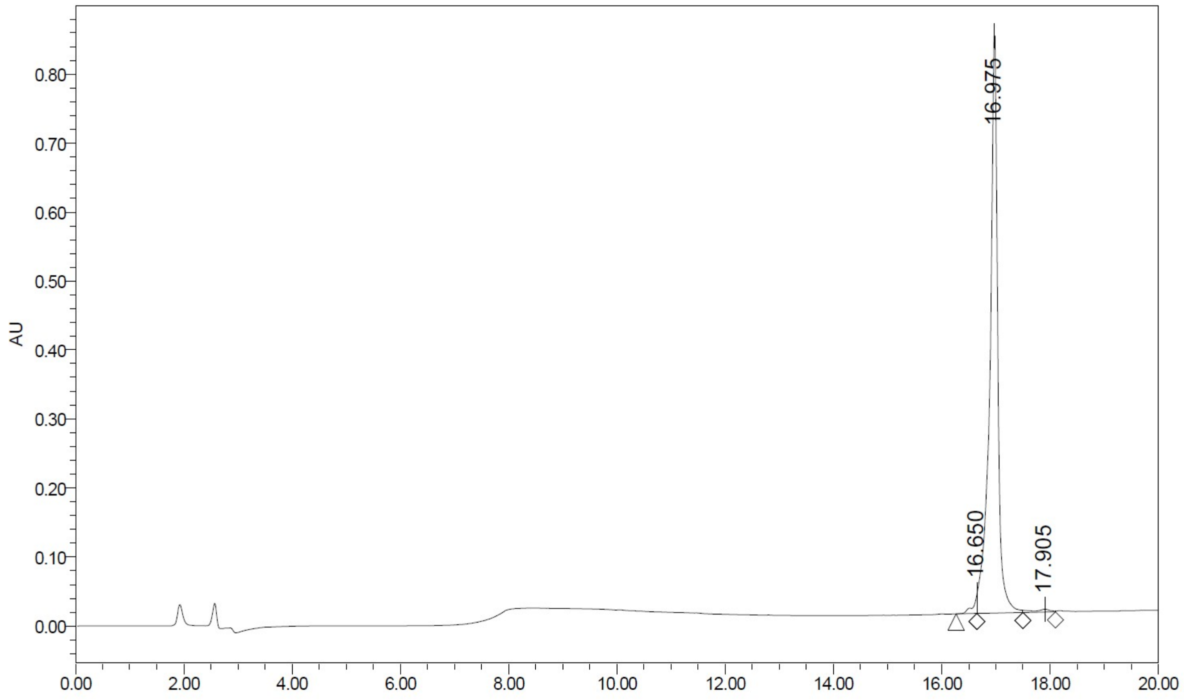

Figure S13. Analytical HPLC of ([Lys^{35}(Ahx-CF)] h- -CGRP after oxidation and purification. Mtt was used for Lys^{35} protection.

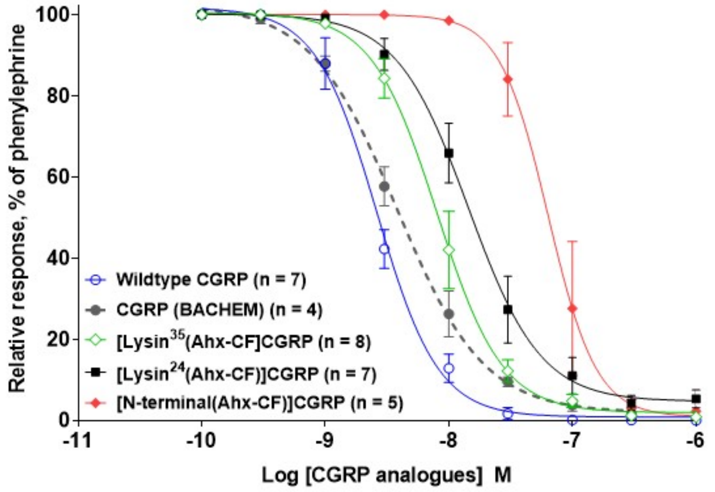

Figure S14. Concentration-response curves with compounds used in this study.

S14: Cumulative concentration-response curves (10 pM – 1 μM) for wild-type CGRP, BACHEM´s CGRP and fluorescently tagged analogues in isolated human subcutaneous arteries. Data points represent mean values and vertical bars indicate ± SEM, where this value exceeds the size of symbol (n = number of animals or number of human subcutaneous arterial ring segments). Relative responses are given as fraction (stated as percentages) of the initial vessel contractile response to 10 μM phenylephrine just before they were challenged with the compounds. Figure shows close similarity between concentration-response curves with CGRP purchased from BACHEM (dashed line with closed circle)and the wild-type CGRP (open circle) synthetized in our laboratory.

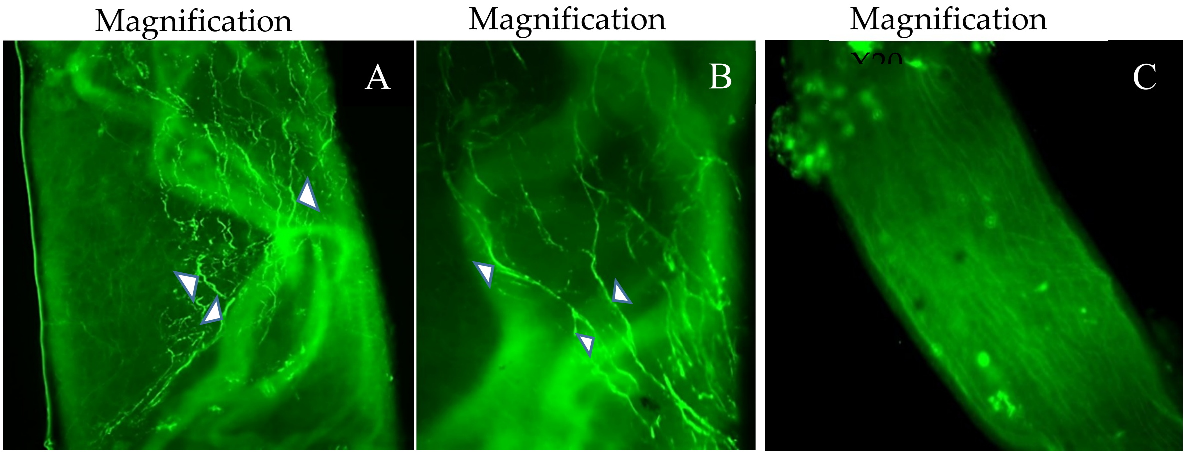

Figure S15. whole-mount staining of a single rat 2nd order mesenteric artery segment with the analogue. [Lys35(Ahx-CF)]-CGRP (A and B). The arterial segments were first challenged by capsaicin (0.1 μM) for 30 minutes and washed in PSS. Afterwards, the arterial segments were depolarized with 125 Mm KPSS for 5 minutes, re-washed in PSS, preconstricted by 60 mM KPSS and incubated with either 1 μM K35 or wildtype CGRP for 30 minutes. White arrow heads show neuronal network located on the surface of arterial segment, which is indicating that the fluorescent analogue is taken up by perivascular nerves. The blurred thick structure behind the neuronal network in A and B is the transparent mesenterium attached to the blood vessel wall. The pictures (with 20X and 40X magnification) were taken using Axiovert Zeiss II microscope equipped with CCD camera and processed by ImageJ. The picture C is the control tissue sample, which is incubated with wild type CGRP. Carl Zeiss cubic filter (Excitation: 493 nm and Emission: 520 nm) was used for visualization.
